# Wavelet Coherence Analysis of Plasma Beta, Alfven Mach Number, and Magnetosonic Mach Number during Different Geomagnetic Storms

**DOI:** 10.1155/2024/1335844

**Published:** 2024-03-11

**Authors:** Ashutosh Giri, Binod Adhikari, Rabin Baral, Chali Idosa Uga, Andres Calabia

**Affiliations:** ^1^Department of Physics, St. Xavier's College, Tribhuvan University, Kathmandu, Nepal; ^2^Department of Physics and Astronomy, Clemson University, Clemson, South Carolina, USA; ^3^Department of Space Science, The University of Alabama in Huntsville, Huntsville, AL, USA; ^4^Department of Physics, College of Natural Science, Jimma University, Jimma, Ethiopia; ^5^Department of Mathematics, Universidad de Alcalá, Alcalá de Henares 28805, Madrid, Spain

## Abstract

We study the variation in plasma beta, Alfven Mach number, and magnetosonic Mach number during different geomagnetic storms of solar cycles 23, 24, and 25. In addition, we employ measurements of the solar wind's flow pressure, proton density, interplanetary magnetic field (IMF) along the *z*-direction (Bz), temperature, velocity, and geomagnetic index SYM-H. Here, the wavelet coherence (WTC) approach of plasma beta, the Alfven Mach number, and the magnetosonic Mach number have been used with the symmetrical H component (SYM-H) index, which are critical indicators of the plasma behavior and magnetic field interactions. A solar CME or, much less severely, a corotating interaction region (CIR), which is formed at the leading edge of a high-speed stream, is the source of the magnetic storm. The key objective of this study is to reveal the possible dependencies of the geomagnetic indices on whether a storm is driven by a CME or CIR. For CIR-associated storms, large amplitude waves occur preferentially with the rising Alfven Mach number and plasma beta. At the same time, the magnetosonic Mach number lacks variability during the storms caused by shock on the arrival of Earth's environment. This is different for CME-driven storms, where the variations of the magnetosonic Mach number do not show much fluctuation compared to the Alfven Mach number and plasma beta. WTC between SYM-H and our derived parameters indicates periodicities between 64 and 512 minutes and noticeable regions of significantly enhanced power on November 07–09, 2004, and June 21–23, 2015. However, the magnetosonic Mach number showed a noticeable coherence with SYM-H between 64 and 250 minutes on September 06–08, 2017. Although, during March 19–21, 2021, both the Alfven Mach number and magnetosonic Mach number showed a noticeable coherence with SYM-H, plasma beta showed none. These parameters can be used in the prediction of geomagnetic storms of the category above G3.

## 1. Introduction

Earth-directed coronal mass ejections (CMEs) have a strong correlation with the incidence of geomagnetic storms. Magnetic energy released by Sun results in CMEs. The energy released is marked by electromagnetic brightening at a variety of wavelengths known as solar flares. A subclass of CMEs known as magnetic clouds has rotations that resemble flux ropes in their magnetic field patterns. One of the many names for CMEs in the solar wind is ejecta [1]. Depending on their dynamical and magnetic features, these CMEs are potentially geoeffective when they reach Earth [2]. The interaction between solar activities and Earth's magnetosphere is caused by magnetic reconnection and viscous phenomena such as Kelvin–Helmholtz instability [3, 4]. When the *z* component of the interplanetary magnetic field (IMF-Bz) > 0 nT is directed northward, such viscous processes play a significant role [5]. When IMF-Bz turns southward (IMF-Bz < 0 nT), which causes magnetic reconnection on the dayside magnetopause to become more responsible for the majority of plasma transport, solar wind couples to Earth's space more efficiently. One of the most reliable signs of a magnetic storm is an enhanced ring current. An abrupt rise in the horizontal component of the geomagnetic field H at several locations is the initial sign of a geomagnetic disturbance. The global magnetic storm intensity is tracked using the disturbance storm time (Dst) index. These are produced by employing a longitudinally distributed chain of low- and mid-latitude ground-based magnetometers which measure ring current strength [6]. The increase of ring current encircling Earth in the equatorial plane is directed from east to west which causes the Dst value to be negative, and this negative value increases with increasing strength of storm [7]. The geomagnetic storms are divided into four categories depending on the value of Dst as suggested as weak or small (−30 nT to −50 nT), moderate (−50 nT to −100 nT), intense (−100 nT to −250 nT), and severe (−250 nT and below) [8]. The symmetrical H component of disturbance (SYM-H) and Dst are comparable indices, and SYM-H has a one-minute temporal resolution, which is beneficial for analyzing brief temporal fluctuations during geomagnetic storms [9].

The planet's magnetosphere serves as an impenetrable barrier to the continuous flow of plasma from Sun [10]. The complex relationships between the solar plasma streams and Earth's magnetic field are dependent on a number of control factors that affect geomagnetic storms. These factors include plasma beta, which characterizes the magnetic cloud, and Mach numbers *M* (ratio of the relative speed to a characteristic wave speed) [11]. The shock's Mach number increases as the differential speed increases. The relative significance of plasma pressure to magnetic pressure in magnetized plasma is described by the dimensionless parameter known as the plasma beta. Variations in plasma beta can affect how plasma waves and instabilities behave during geomagnetic storms and play a crucial role in determining the character of plasma dynamics. Plasma beta is the ratio of thermal energy density and magnetic energy density.

Another essential component is the Alfven Mach number, which expresses the relationship between the plasma flow speed and the Alfven speed, or the rate at which magnetic disturbances move through magnetized plasma. The Alfven Mach number (*M*_*A*_), given as *V*_sw_/*V*_*A*_, characterizes the strength of the magnetic field (where *V*_sw_ is the velocity of plasma flow and *V*_*A*_ is the Alfven speed: the speed with which hydrodynamic waves propagates) [12]. Changes in the Alfven Mach number can reveal information about how effectively energy is transferred from the solar wind to the magnetosphere of Earth during geomagnetic storms. *M*_*A*_ (<1) corresponds to a strong magnetic field termed as sub-Alfvenic and *M*_*A*_ (>*r*bin1) conditions correspond to a weak magnetic field termed as super Alfvenic [13, 14]. The Alfven and sound speeds in magnetized plasma are combined to create the magnetosonic speed, which is a composite speed that is represented by the magnetosonic Mach number. It describes the impact of compressibility on the plasma and can provide important details on how fast and slow magnetosonic waves spread during geomagnetic disturbances.

The average interplanetary quiet field is 3–8 nT, and shock compression (magnetic field jump) across the shock of this field is roughly proportional to the Mach number. The low beta values (≈0.1) in clouds imply large Alfven/magnetosonic speeds which would ordinarily preclude the formation of shocks within magnetic clouds [15]. The authors of [16] discovered that there are distinct non-Gaussian statistics in the directions perpendicular to the magnetic field for a very high Alfvenic Mach number and high plasma beta. On the other hand, the kurtosis is modest, and the plasma is nearly at equilibrium for directions parallel to this field [17]. Furthermore, when the solar wind *M*_*A*_ is high, then thermal plasma forces dominate, but when it is low, magnetic forces dominate.

Because of the complexity and variability of Earth's magnetosphere and solar wind, solar wind structures produce storms of varying sizes. The size and intensity of geomagnetic storms are influenced by a number of factors, including solar wind speed, density, magnetic field orientation, composition (including solar flare and coronal mass ejection presence), Earth's magnetic field strength, and variability in the solar cycle [18, 19]. The variety in storm magnitude seen in response to various solar wind configurations is the result of complex interactions between these elements. Because of this, it is still difficult to forecast the exact size of a geomagnetic storm that would arise from a given solar wind event, and the result might vary greatly based on how these elements interact. Therefore, in this study, we employed the wavelet coherence approach to analyze the variations in plasma beta, Alfven Mach number, and magnetosonic Mach number during selected geomagnetic storms of solar cycles 23, 24, and 25. By examining the coherence and phase relationships between these plasma parameters and the SYM-H index, we aim to gain deeper insights into the underlying physical processes driving these variations.

## 2. Datasets and Methods

. In this work, we used data from OmniWeb (https://omniweb.gsfc.nasa.gov/) maintained by the Space Physics Data Facility at the NASA/Goddard Space Flight Center during four geomagnetic storms that took place during solar cycles 23, 24, and 25. Alfven Mach number, magnetosonic Mach number, plasma beta, flow pressure, proton density, solar wind speed (Vsw), plasma temperature (Tsw), z-component of interplanetary magnetic field (IMF-Bz), and H component of the symmetric ring current index (SYM-H) were the parameters we chose from the OMNI system. We chose these events because of their different intensities to find the coherence pattern between Mach numbers, plasma beta, and SYM-H. The reason to choose intense and super intense storms is to check the possible coherence between a pattern and one quiet event for comparison and to study the variation of Mach numbers and plasma beta in weak storms. The SYM-H index was used to select these events. We classified these events as weak, intense, and super intense storms as shown in Table 1.

In this work, we studied the time series analysis of four events to uncover important details of the dynamics of the geomagnetic storm and magnetosphere. Furthermore,wavelet coherence (WTC) analysis was used to find the phase relation between the plasma beta, Alfven Mach number, and magnetosonic Mach number with SYM-H. The primary driving forces behind such research in studies like this are pattern identification and forecasting. After implementing time series analysis, a more powerful tool known as wavelet analysis is used. Wavelet analysis can analyze nonstationary signals and the confined oscillation feature in time-frequency space [20, 21].

The two types of wavelet analysis are continuous wavelet transform (CWT) and discrete wavelet transform (DWT). The Morlet wavelet (dimensionless frequency, *ω*_*o*_ = 6) is an excellent choice for feature extraction for its adequate time and frequency localization [21]. These wavelet transforms have been expanded to a bivariate wavelet analysis: cross wavelet (XWT) and wavelet coherence (WTC) that investigate the time-frequency domain correlation between two signals for determining the phase angle between the time series [22]. The XWT of two-time series *X* (*t*) and *Y* (*t*) is defined as

(1) $$\begin{array}{c} W_{X,Y}\left(a,b\right)=W_{X}\left(a,b\right)W_{Y}^{*}\left(a,b\right), \end{array}$$

where *W*_*X*,*Y*_(*a*, *b*) is the cross-wavelet power spectrum, the sequence *X* (*t*) wavelet transform coefficients are denoted by *W*_*X*_(*a*, *b*), and the complex conjugate of the sequence *Y* (*t*) wavelet transform coefficients is denoted by *W*_*Y*_^*∗*^(*a*, *b*). Furthermore, *a* and *b* are the scaling and time translation parameters, respectively.

The WTC spectrum is used to determine how coherent the cross-wavelet transform is in time-frequency space with more effectiveness at identifying time-frequency correlations even in areas where both time series have low powers compared to XWT [23]. The cross-wavelet coherence spectrum was defined by [21] as follows:

(2) $$\begin{array}{c} R^{2}\left(a,b\right)=\frac{{\left(S\left[a^{-1}W_{XY}\left(a,b\right)\right]\right)}^{2}}{S\left[a^{-1}W_{X}\left(a,b\right)\right]S\left[a^{-1}W_{Y}\left(a,b\right)\right]}, \end{array}$$

*S* is the smoothing operator in this case, and it is given by [21]

(3) $$\begin{array}{c} S\left(W\right)=S_{\text{scale}}\left(S_{\text{time}}\left(W_{X,Y}\left(a,b\right)\right)\right), \end{array}$$

where *S*_scale_ and *S*_time_ denote smoothing along the wavelet scale axis and smoothing in time, respectively [20, 21, 23]. WTC values around 1 indicate a higher degree of resemblance across time series, while coherence values near 0 indicate no correlation [24].

## 3. Result and Discussion

In this section, we implemented a time series analysis of selected parameters for pattern identification, followed by WTC to find the phase relation between the plasma beta, Alfven Mach number, and magnetosonic Mach number with SYM-H.

### 3.1. Event 1: Super Intense Storm (November 08, 2004)

**Figure 1:**
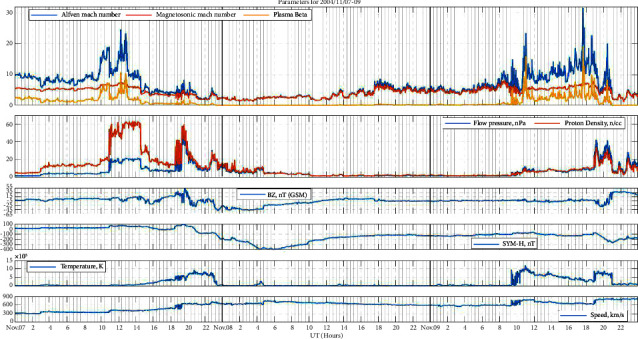
Space weather and geomagnetic indices during the event of 7th–9th November 2004 each day separated by vertical black lines. From the top panel to the bottom panel, the top panel: Alfven Mach number, magnetosonic Mach number, and plasma beta, the second panel: flow pressure and proton density, the third panel: IMF and Bz, the fourth panel: SYM-H, the fifth panel: temperature, and the sixth panel: solar wind velocity.

In Figure 1, the top panel shows the Alfven Mach number, magnetosonic Mach number, and plasma beta, the second panel shows flow pressure and proton density, IMF and Bz are in the third panel, SYM-H and temperature are in the fourth and fifth panels, respectively, and the sixth panel shows solar wind velocity. On November 7, there was an abrupt change in the Alfven Mach number (≈25) and plasma beta (≈10) around 10:00–14:00 followed by a change in proton density (≈60 n/cc) and flow pressure (≈20 nPa), and just after 11:00 UT, the first cluster of geomagnetic activity began to emerge. At least three sudden impulses (SIs) and several rapid IMF–Bz shifts were present as the sheath of the initial ICME approached Earth's magnetosphere. During the next hours, strong geomagnetic activity developed rapidly as the IMF went southward (Bz≈−20 nT around 14:00–16:00 UT) and decayed when the IMF turned northward (Bz≈+50 nT around 16:00–22:30 UT). During this period, there were no sharp changes in the Alfven Mach number and plasma beta; however, the magnetosonic Mach number showed some rapid changes compared to previous hours. The solar wind was largely undisturbed between 18:00 UT on November 8 and 10:00 UT on November 9, but afterward till 21:00 UT, the Alfven Mach number (>*r*bin 25) and plasma beta (≈20) were changing rapidly The second significant storm began about 19:00 UT November 9 when the Alfven Mach number (>*r*bin 25) and plasma beta (≈20) were at peak as the second ICME began to stream across the magnetosphere. On this day, a distinct short-duration spike in the geomagnetic field was caused by a significant negative excursion in IMF-Bz just after 20:00 UT.

### 3.2. Event 2: Intense Storm (June 22, 2015)

**Figure 2:**
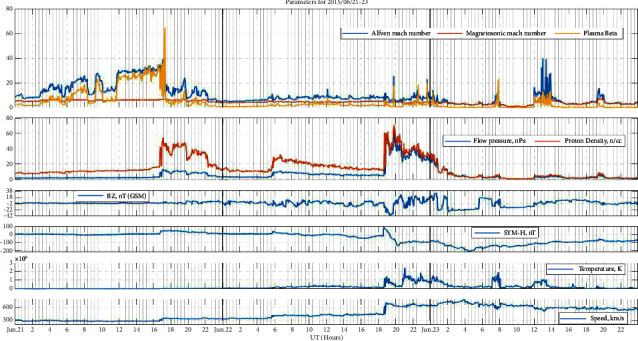
Space weather and geomagnetic indices during the event of 21st–23rd June 2015, each day separated by vertical black lines. From the top panel to the bottom panel, the top panel: Alfven Mach number, magnetosonic Mach number, and plasma beta, the second panel: flow pressure and proton density, the third panel: IMF Bz, the fourth panel: SYM-H, the fifth panel: temperature, and the sixth panel: solar wind velocity.

From Figure 2, in a similar manner, three interplanetary shocks passed the L1 point between June 21 and 22, according to the SOHO CELIAS/MTOF Proton Monitor on the SOHO satellite [25]. The initial one was discovered on June 21 around 16:00 UT. The weak impact with Earth's magnetic field was seen at ≈17:00 UT just after plasma beta and Alfven Mach number returned to their lowest value ≈10 and ≈18 from peak values ≈65 and ≈40, respectively. However, there was no immediate geomagnetic storm as a result of this hit. On June 22, about 05:00 UT, a second interplanetary shock crossed L1, boosting the solar wind's velocity and density; a third interplanetary shock followed around 18:00 UT, as seen in Figure 2. The second impact compressed Earth's magnetosphere, while the Bz magnetic component oscillated over time. At 19:00 UT, the third impact on Earth was detected, resulting in a modest rise in the Alfven Mach number and plasma beta. After the third impact, there was a prolonged, primarily southbound Bz phase that persisted from 02:00 UT on June 23 until 06:00 UT that day, dropping as low as −25 nT. The authors of [25] named this G4 solstice storm the 2015 Summer Solstice Storm based on the superposition effects of a series of passing ICMEs and their repercussions in Earth's magnetic field and adjacent areas (such as the L1).

### 3.3. Event 3: Intense Storm (September 07, 2017)

**Figure 3:**
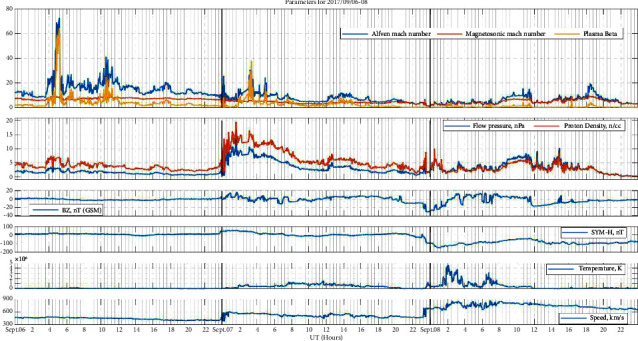
Space weather and geomagnetic indices during the event of 6th–8th September 2017, each day separated by vertical black lines. From the top panel to the bottom panel, the top panel: Alfven Mach number, magnetosonic Mach number, and plasma beta, the second panel: flow pressure and proton density, the third panel IMF Bz, the fourth panel: SYM-H, the fifth panel: temperature, and the sixth panel: solar wind velocity.

Proceeding in a similar manner, Figure 3 shows the extreme solar activity in early September 2017 at a minimum of solar cycle 24. At 23:00 UTC on September 7, 2017, a coronal mass ejection (CME) caused by the X9.3 solar flare on September 6, 2017, arrived on our planet. In G3, strong geomagnetic storming started at 23:25 UT and became severe at 23:50 UT (Kp = 8) in G4 [26, 27]. According to [28], an evident shock ICME complex structure was the primary source of this severe geomagnetic storm. Here, in this event, the plasma beta and Alfven Mach number lag behind the pressure and density as we can see from the figure. The plasma beta and Alfven Mach number were at peak reaching the highest value among all events, ≈60 and ≈70, respectively, around 18 hours before the event. A powerful geomagnetic storm with a peak Dst value (Dst min) of −142 nT occurred on September 8, 2017, at 02:00 UT.

### 3.4. Event 4: Weak Storm (March 20, 2021)

**Figure 4:**
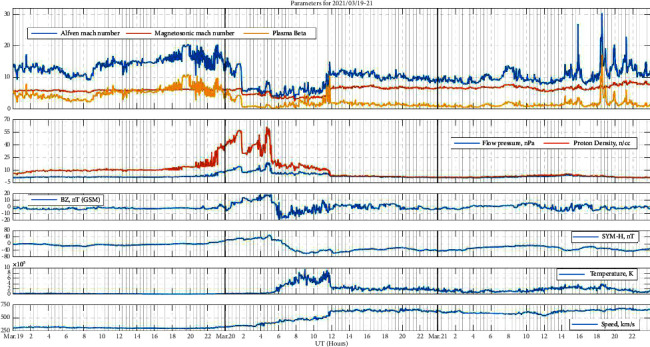
Space weather and geomagnetic indices during the event of 19th–21st March 2021, each day separated by vertical black lines. From the top panel to the bottom panel, the top panel: Alfven Mach number, magnetosonic Mach number, and plasma beta, the second panel: flow pressure and proton density, the third panel: IMF Bz, the fourth panel: SYM-H, the fifth panel: temperature, and the sixth panel: solar wind velocity.

From Figure 4, it is clearly seen that this is a weak storm accompanied by a high-speed stream as we can see speed is increasing constantly from 2UT hours of March 20 along with other parameters. The storm is linked to the CIR ahead of the stream, specifically to Bz's initial northward turn in the leading portion of the CIR and subsequent southbound turn in the following half, which creates this weak storm. No significant changes can be seen in the plasma beta, Alfven Mach number, or magnetosonic Mach number before the event day.

### 3.5. WTC Results

Each panel of the figures exhibits the WTC of plasma beta and SYM-H, Alfven Mach number and SYM-H, and magnetosonic Mach number and SYM-H, respectively. The vertical axis is the period; the color depth represents the magnitude of coherence between plasma Beta—SYM-H, Alfven Mach number—SYM-H, and magnetosonic Mach number—SYM-H, and the arrow represents the phase difference between two parameters; ⟶ indicates that the two are in the same phase or positively correlated; ← indicates that the two are in the opposite phase or inversely related; ↓ and ↑ indicate that the latter lags behind and leads the former by 90^*o*^ [29]. The *x*-axis represents the time period. The thick black contours depict the wavelet power spectrum's 95 % confidence level after Monte Carlo simulations using a phase-randomized surrogate series [21]. The other regions (the gray-shaded areas) are cones of interference where edge effects may impact the accuracy of the analysis; this is placed to divide regions with reliable and inaccurate estimations. Power is denoted by a color code that ranges from blue (low power) to yellow (high power) [30].

**Figure 5:**
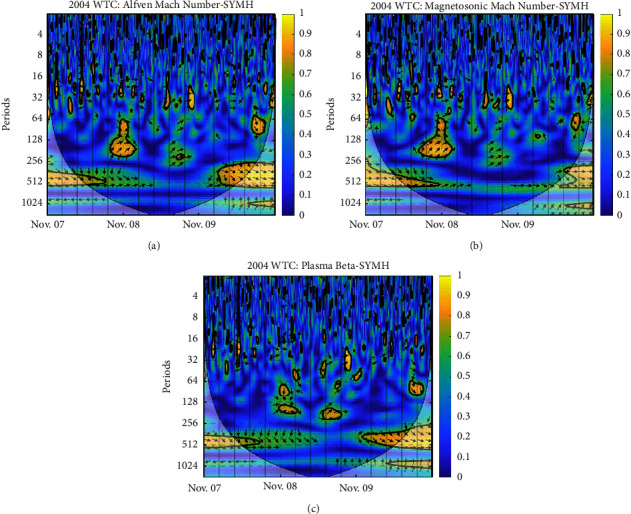
WTC between SYM-H and derived parameters: (a) Alfven Mach number; (b) magnetosonic Mach number; (c) plasma beta on 2004 November 07–09.

From Figure 5, plasma beta has 4 considerable regions of higher power at a periodicity of nearly 64 minutes, and there are 2 noticeable regions of higher power on Nov 08-09. At the beginning of Nov 08, SYM-H lags behind plasma beta by 90° which shows the commencement of a storm; then around the end of Nov 09, SYM-H lags behind by some certain angle. At a periodicity of 128 minutes, we can clearly see plasma beta and SYM-H are in phase in the early and end hours of November 08, and SYM-H is leading plasma beta with a minimal angle. In the case of the Alfven Mach number and magnetosonic Mach number, 2 strong regions of higher power can be seen from periodicity 64–230 minutes on Nov 08 to 64–120 minutes on Nov 09. Clearly, both Mach numbers show the same phase relation pattern with SYM-H first around 64 periodic parts; SYM-H lags behind both the Mach numbers, being at the same phase around 128 minutes periodicity and then leading those Mach numbers with some angle.

**Figure 6:**
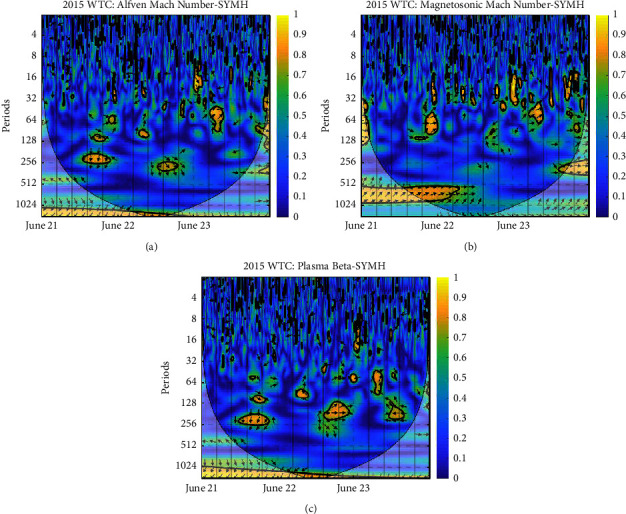
WTC between SYM-H and derived parameters: (a) Alfven Mach number; (b) magnetosonic Mach number; (c) plasma beta on 2015 June 21–23.

From Figure 6, no considerably strong correlation is seen in the case of the magnetosonic Mach number except at the end of June 21 at a periodicity of 64 minutes where the magnetosonic Mach number had the same phase as SYM-H. In the case of plasma beta and Alfven Mach number, regions of higher power exist at periods of 128 and 256 minutes around the end of June 21, 2015, with the same phase pattern as SYM-H which is in phase with plasma beta and Alfven Mach number around 128 minutes periodicity with a very small coherence region. At the end of June 22, SYM-H lags behind plasma beta and Alfven Mach number with 256 minutes periodicity, and around the 128 minutes period, SYM-H leads plasma beta with some angle; then, they attain the same phase around 200 minutes periodicity. While in the case of the Alfven Mach number around the end of June 22, SYM-H lags behind the Alfven Mach number. Strange regions of higher power are seen in the case of plasma beta at the end of this storm, i.e., June 23 around periodicity 200 minutes where plasma beta and SYM-H show opposite phases.

**Figure 7:**
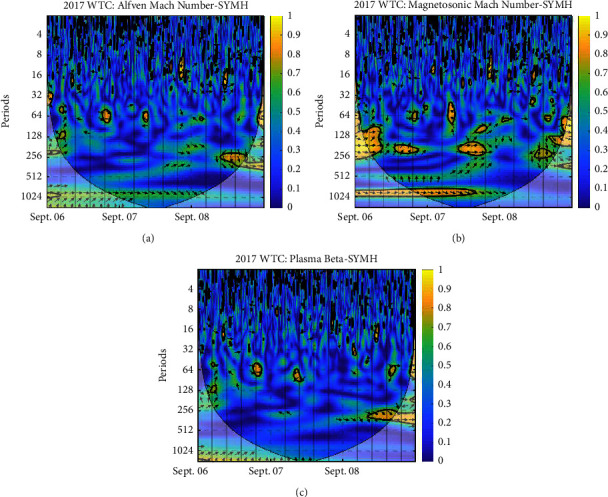
WTC between SYM-H and derived parameters: (a) Alfven Mach number; (b) magnetosonic Mach number; (c) plasma beta on 2017 September 06–08.

In the storm of 2017 September 06–08, Figure 7 depicts no strong relationship between plasma beta and SYM-H; the same is the case of the Alfven Mach number and SYM-H, but the magnetosonic Mach number shows a strong correlation with SYM-H at the periodicity of 200–256 minutes. Around the end of Sept 06, SYM-H leads the magnetosonic Mach number; again at around the end of Sept 07, they are in phase, and at the end of Sept 08, SYM-H lags behind the magnetosonic Mach number. No considerable relation can be extracted through this plot as this is a storm caused by high-speed steamers.

**Figure 8:**
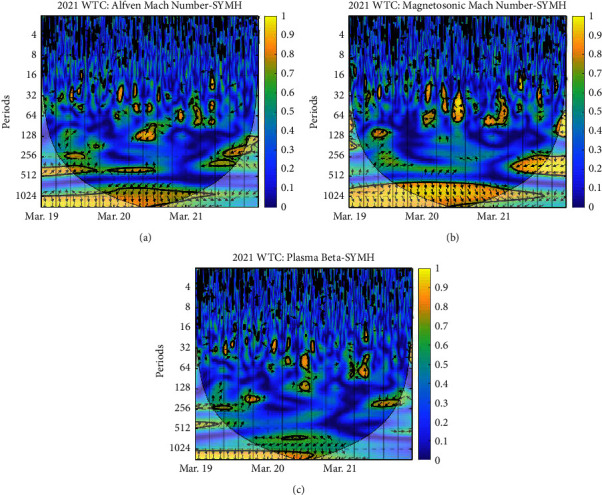
WTC between SYM-H and derived parameters: (a) Alfven Mach number; (b) magnetosonic Mach number; (c) plasma beta on 2021 March 19–21.

Figure 8 shows on March 19–21, 2021, both the Alfven Mach number and the magnetosonic Mach number showed noticeable regions of higher power with the SYM-H index throughout the event with a greater periodicity. However, in the case of plasma beta, no significant phase relation can be drawn from the coherence region.

## 4. Conclusion

The geoeffectiveness of a CME and its time of arrival on Earth are the most crucial aspects of any prediction. In this research, Wavelet analysis has been used in order to understand the relationship between plasma beta, Alfven Mach number, and magnetosonic Mach number with SYM-H. We applied this approach to 4 different events of solar cycles 23, 24, and 25. The major findings of this research are as follows:

1. Based on real-time data, an analysis of the space weather conditions for the November 2004 event reveals the initial complexity of the solar sources as well as the continuing interactions of CMEs as they propagate through the solar wind. Only two complicated geoeffective ICME complexes were formed as a result of these interactions, and they reached Earth on November 7–8 and 9–10. Our result shows that a rise in the Alfven Mach number can be a sign that the solar wind contains rapid flows or shock waves. Therefore, Mach numbers can be used to predict severe storms caused by CMEs and ICMEs.
2. Magnetic reconnection, which occurs when magnetic field lines connect and release energy from various locations, is significantly influenced by plasma beta. This explains the sudden increase in plasma beta for events 1, 2, and 3 which were super intense and intense storms. Geomagnetic disruptions may be brought on by this energy transfer. High plasma beta can be used to identify areas with a higher probability of reconnection. Monitoring variations in plasma beta can reveal information about the likelihood of magnetic reconnection events and their potential role in the development of geomagnetic storms.
3. Wavelet coherence (WTC) analysis of geomagnetic storms reveals distinct patterns of coherence and phase relationships across a spectrum of frequencies, underscoring dynamic coupling between solar wind conditions and geomagnetic responses. During super intense storms, notably on November 8, high coherence is observed at lower periodicities (64 and 128 minutes), demonstrating in-phase and leading-lagging relationships between plasma beta and the SYM-H index, as well as strong correlations with Alfven and Magnetosonic Mach numbers, especially within the 64 to 230 minutes range. For the intense storm of June 21, 2015, WTC points to specific periods where plasma beta and Alfven Mach number closely align with SYM-H at 128 and 256 minutes periodicities. Conversely, the September 6–8, 2017, storm exhibits a pronounced correlation between the magnetosonic Mach number and SYM-H at 200–256 minutes. During a weak storm (March 19–21, 2021), significant interactions between Alfven and magnetosonic Mach numbers with SYM-H are detected, whereas plasma beta shows a negligible phase correlation.
4. These findings highlight the nuanced influence of geomagnetic storm intensity on solar wind-geomagnetic interactions, with low periodicity in super intense storms indicating a heightened frequency of coupling between Mach numbers, plasma beta, and SYM-H. The frequency decreased as the intensity of the geomagnetic storm decreased.

In conclusion, although plasma beta, Alfven Mach number, and magnetosonic Mach number are not capable of reliably predicting geomagnetic storms on their own, they are extremely important for understanding the underlying physical mechanisms that underlie space weather phenomena. We can better predict and reduce the effects of geomagnetic storms on technological systems and satellite operations by including these factors in prediction models and using them as a part of an extensive monitoring approach. With further work using different tools and techniques and large datasets, these results can be used in predicting moderate to severe geomagnetic storms.

## Figures and Tables

**Table 1:** Selected event days with SYM-H values and storm types.

S.N.	Event days	SYM-H value (nT)	Storm type
1	Event 1: 2004 November 07–09	−373	Super-intense
2	Event 2: 2015 June 21–23	−195	Intense
3	Event 3: 2017 September 06–09	−122	Intense
4	Event 4: 2021 March 19–21	−45	Weak

## Data Availability

The space weather data are available from the website of High Resolution OMNI-OMNIWeb Data Explorer-NASA (https://omniweb.gsfc.nasa.gov/form/omni_min.html).
